# *BAP1* Missense Mutation c.2054 A>T (p.E685V) Completely Disrupts Normal Splicing through Creation of a Novel 5’ Splice Site in a Human Mesothelioma Cell Line

**DOI:** 10.1371/journal.pone.0119224

**Published:** 2015-04-01

**Authors:** Arianne Morrison, Yvonne Chekaluk, Ruben Bacares, Marc Ladanyi, Liying Zhang

**Affiliations:** 1 School of Medicine, Wake Forest University, Winston Salem, North Carolina, United States of America; 2 Department of Pathology, Memorial Sloan Kettering Cancer Center, New York, New York, United States of America; International Centre for Genetic Engineering and Biotechnology, ITALY

## Abstract

*BAP1* is a tumor suppressor gene that is lost or deleted in diverse cancers, including uveal mela¬noma, malignant pleural mesothelioma (MPM), clear cell renal carcinoma, and cholangiocarcinoma. Recently, *BAP1* germline mutations have been reported in families with combinations of these same cancers. A particular challenge for mutation screening is the classification of non-truncating *BAP1* sequence variants because it is not known whether these subtle changes can affect the protein function sufficiently to predispose to cancer development. Here we report mRNA splicing analysis on a homozygous substitution mutation, *BAP1* c. 2054 A&T (p.Glu685Val), identified in an MPM cell line derived from a mesothelioma patient. The mutation occurred at the 3rd nucleotide from the 3’ end of exon 16. RT-PCR, cloning and subsequent sequencing revealed several aberrant splicing products not observed in the controls: 1) a 4 bp deletion at the end of exon 16 in all clones derived from the major splicing product. The *BAP1* c. 2054 A&T mutation introduced a new 5’ splice site (GU), which resulted in the deletion of 4 base pairs and presumably protein truncation; 2) a variety of alternative splicing products that led to retention of different introns: introns 14–16; introns 15–16; intron 14 and intron 16; 3) partial intron 14 and 15 retentions caused by activation of alternative 3’ splice acceptor sites (AG) in the introns. Taken together, we were unable to detect any correctly spliced mRNA transcripts in this cell line. These results suggest that aberrant splicing caused by this mutation is quite efficient as it completely abolishes normal splicing through creation of a novel 5’ splice site and activation of cryptic splice sites. These data support the conclusion that *BAP1 c*.*2054 A&T* (p.E685V) variant is a pathogenic mutation and contributes to MPM through disruption of normal splicing.

## Introduction

*BAP1* is a tumor suppressor gene that is lost or deleted in diverse cancers, including uveal melanoma,[1] malignant pleural mesothelioma (MPM),[2] clear cell renal carcinoma,[3] and cholangiocarcinoma.[4] Recently, *BAP1* germline mutations have been reported in families with combinations of these same cancers. [5,6] It has been proposed that *BAP1* germline mutations define a new familial cancer syndrome. [7,8]

*BAP1* is located on chromosome 3p21 and contains 17 coding exons. The BAP1 protein is composed of 729 amino-acids. BAP1 is a nuclear ubiquitin carboxy terminal hydrolase (UCH), a subfamily of deubiquitinating enzymes, which was initially identified as a protein that bound to the RING finger domain of BRCA1. [9] The interaction requires a wild-type BRCA1-RING finger as BAP1 does not bind to germline mutants of the BRCA1-RING finger found in patients with hereditary breast and ovarian cancers. [9] In addition to the UCH catalytic domain and BRCA1 interacting domain, BAP1 contains a UCH37-like domain (ULD), binding domains for BARD1, and a binding domain for HCFC1. The interaction with BRCA1 and BARD1 form a tumor suppressor heterodimeric complex. [10] BAP1 regulates cell proliferation and interacts with histone-modifying complexes during cell division. [11,12] BAP1 also forms the Polycomb group repressive deubiquitinase complex (PR-DUB) through interaction with ASXL1, which is involved several developmental processes. [13] Because of this functional complexity, *BAP1* germline mutations predispose individuals to the aggressive tumor phenotypes.

It is relatively straightforward to interpret germline *BAP1* truncating mutations (nonsense mutation, small insertions/deletions) and alterations of a canonical dinucleotide splice donor/acceptor sequences that affect the GU-AG rules. Classification of some non-truncating sequence variants in tumor suppressor genes can be problematic because it is not known whether these subtle changes alter function sufficiently to predispose to cancer development. As a result, carriers of variants of unknown significance and their family members cannot take advantage of the risk assessment, prevention, and therapeutic measures that are available to carriers of known pathogenic mutations.

Traditionally, to determine the pathogenic effect of a substitution variant, the focus is placed on its effects on protein structure and function. However, single nucleotide substitutions within exons can also have significant impact on mRNA processing and thereby, on protein function.[14] Since normal splicing of pre-mRNAs is an essential step in protein expression, the potential to disrupt this step should always be investigated for substitution mutations and synonymous mutations that may affect the authentic splice sites or disrupt exonic splicing enhancers (ESEs), [15] especially for variants that affect the last nucleotide of an exon. [16]

Here we report a homozygous substitution mutation, *BAP1* c. 2054 A>T (p.Glu685Val) in exon 16 in an MPM cell line, HMeso01A, identified through *BAP1* full gene sequencing analysis.[2] Since the missense mutation affects the 3^rd^ nucleotide at the 3’ end of exon 16 (the -3 position), we investigated whether it had any effect on mRNA splicing. We performed RT-PCR and cloned the products into the sequencing vectors. We identified multiple splicing variants that lacked exons 14–17 of the wild type *BAP1* sequence. We demonstrated that this mutation creates a novel 5’ splicing site and completely disrupts normal splicing of *BAP1* at this splicing site. This mutation mainly introduced a 4 base pair deletion, which presumably leads to a truncated protein. We also observed retention of neighboring introns in a relatively small fraction of the aberrant splicing products. Our study indicated that, instead of introducing a missense mutation in the mature protein, some substitution mutations outside of core splice sites may nonetheless contribute to tumorigenesis through disruption of normal splicing.

## Materials and Methods

### Comparative genomic analysis

Sequence data spanning 10.5 kb of the *BAP1* locus for Homo sapiens [chromosome 3, position 52 400 413–52 410950, Ensembl release 78—December 2014] was obtained from the Ensembl Genome Browser (http://www.ensembl.org/index.html). Pair-wise sequence comparisons were carried out using the Ensembl Genomic alignments function. *BAP1* cDNA and protein sequences of selected species were selected for multiple sequence alignment using ClustalW2 (http://www.ebi.ac.uk/Tools/msa/clustalw2/). For these comparisons, Homo sapiens was considered the base sequence.

### *In silico* analysis

Three commonly used programs were used to predict the potential effects of amino acid substitutions on protein structure and activity: PolyPhen (Polymorphism Phenotyping, http://genetics.bwh.harvard.edu/pph2/), [17] SIFT (Sorting Intolerant from Tolerant, http://sift.jcvi.org/), [18] and MutationTaster (http://www.mutationtaster.org/). [19] Splicing Regulation Online Graphical Engine (Sroogle, http://sroogle.tau.ac.il/) was used to predict the potential effects on normal mRNA. Scroogle provides a comprehensive platform that allows visualization of potential splice signals (5’ and 3’ splice sites (5’ss and 3’ss), the polypyrimidine tract located upstream of the 3’ss and the branch site) and the splicing-regulating sequences in a sequence of interest in an integrated, user-friendly and easily interpretable format. [20] The percentile scores are calculated based on the distribution of values for each of these signals within each of these two datasets (the 50, 000 constitutively spliced exons and the 3000 alternatively spliced exons, all from the human genome and based on EST data). [21] The percentile score for a user entered sequence indicates the ranking of the user’s sequence within these two pre-calculated distributions. Thus, a value of 0.95 indicates that 95% of the exons have lower scores and only 5% have higher ones. Since Scroogle does not included NNSplice (Splice Site Prediction by Neural Network, http://www.fruitfly.org/seq_tools/splice. html), [22] prediction using NNSplice was performed separately.

### Statistical analysis

Two-tailed unpaired Student's *t*-test was used to perform statistical analysis. In all analysis *p*<0.05 was required for statistical significance.

### cDNA analysis

*BAP1* full gene sequencing identified a homozygous missense mutation, c. 2054A>T Glu685Val, in cell line HMeso01A that was derived from a malignant pleural mesothelioma patient. [23] Total RNA was extracted using the Trizol RNA reagent (Invitrogen Life Technologies, Carlsbad, CA) and was subsequently used for cDNA synthesis (Superscript III First-Strand Synthesis SuperMix, Invitrogen Life Technologies, Carlsbad, CA). Control RNAs were extracted from four individuals seen at Memorial Sloan Kettering Cancer Center who do not carry the c.2054A>T mutation. *BAP1* exons 14–17 were amplified, the sequence of the forward primer is 5’-AGAGAGAAGACGGGGATGGT- 3’ and the sequence of the reverse primer is 5’-TACTGGGAAAAGGGGAAGTG -3’. Each PCR reaction contained 25ul SIGMA REDTaqReady Mix 1x, 2ul of 100ng/ul forward and reverse primers, 2ul of cDNA and water to a final volume of 50ul. Cycling conditions were 96°C for 5min, 94°C for 30 sec (35x), 55°C for 45 sec (35x), and 72°C for 60 sec (35x), with a final extension at 72°C for 5 min. PCR products were separated on a 0.8% SeaKem GTG agarose gel and all bands from cell line HMeso01A were excised separately from the gel and purified (Qiaquick Gel Extraction Kit, Qiagen)

### Cloning

To determine if the mutant allele created alternative transcripts, the gel purified RT-PCR products were cloned into pCR4 TOPO vectors (Invitrogen, Carlsbad, CA), following manufacturer procedures (Invitrogen, Carlsbad, CA). DNA from colonies was amplified using the *BAP1* primers covering cDNA regions of exons 14–17 and subjected to direct DNA sequencing analysis using the forward PCR primer (BigDye Terminator v3.1 Cycle Sequencing kit and 3730 DNA analyzer, Applied Biosystems, Foster City, CA).

## Results

**Fig 1:**
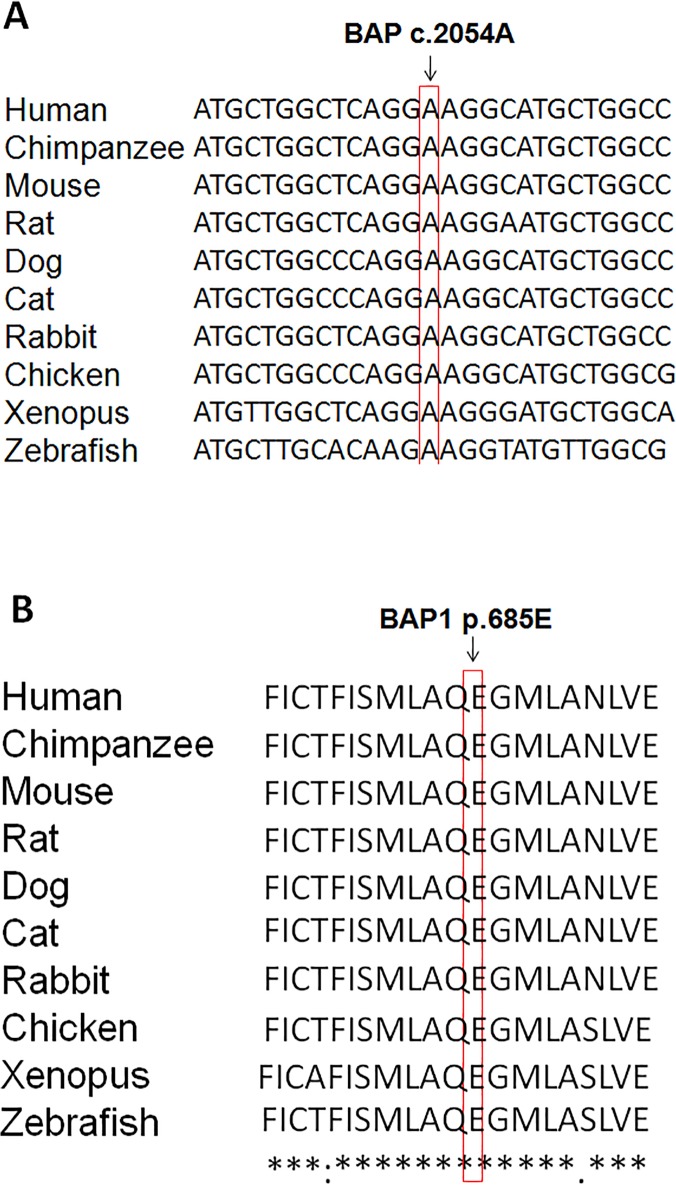
Comparative sequence analysis reveals the *BAP1 c*.*2054A* (p.E685) is highly conserved. A. Multi-species comparative genomic analysis of *BAP1 c*.*2054A* in ten distantly related species: human, chimpanzee, mouse, rat, dog, cat, rabbit, chicken, xenopus and zebrafish. B. Multiple sequence alignment indicates BAP1 p.E685 is well conserved across the same ten distantly related species.

Multi-species comparative genomic analysis was used to identify sequence homology at the *BAP1 c*.*2054 A>T* (p.E685V) mutation site in ten distantly related species: human, chimpanzee, mouse, rat, dog, cat, rabbit, chicken, xenopus and zebrafish. This analysis indicated the *BAP1 c*.*2054A* (p.E585) is highly conserved at both DNA and protein levels across these species (Fig. 1A and B). *In silico* analyses using Polyphen, SIFT and Mutation Taster predicted it to be “probably damaging”, “damaging” and “disease causing” respectively.

**Fig 2:**
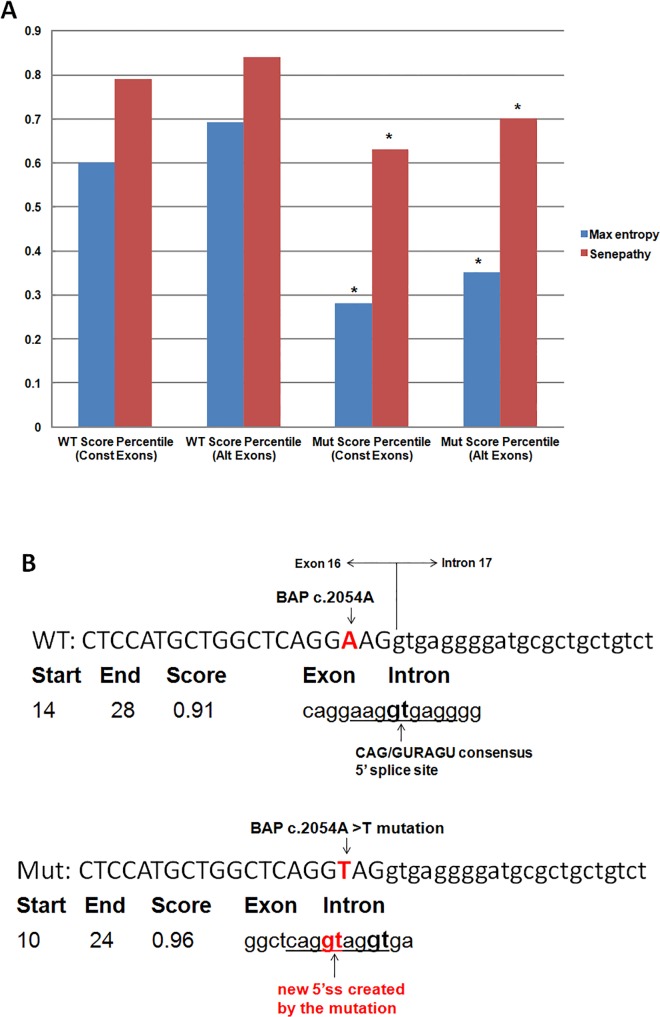
Prediction of the potential effects of the *BAP1 c*.*2054A* (p.E685) alternation on normal mRNA splicing. A. Using Sroogle, the scores of the 5’ splice site in the mutant sequence is significantly reduced for both constitutively spliced exons and alternatively spliced exons based on predictions using Max entropy (*p*<0.02) and the program developed by Senapathy et al. (*p*<0.05). B. NNSplice predicted that the mutation created a strong, new 5’ splice site that matches with the CAG/GURAGU consensus 5’ splice site.

Given the fact that the *BAP1 c*.*2054 A>T* (p.E685V) mutation affects the 3^rd^ nucleotide from the end of exon 16 (-3 position) and the -3 base pair is involved in the U1snRNP binding and initiation of spliceosome assembly, we used Sroogle and NNSplice to predict its potential effects on normal mRNA splicing. As shown in Fig. 2A, the scores of the 5’ splice site in the mutant sequence is significantly reduced for both constitutively spliced exons and alternatively spliced exons based on predictions using Max entropy [24] (t-test, *p* < 0.02) and the program developed by Senapathy [25] (t- test, *p* < 0.05). More interestingly, NNSplice predicted that the mutation created a strong, new 5’ splice site 4 bp upstream of the original one. The new 5’ splice site, if used, will introduce a 4 bp deletion at the end of exon 16 of *BAP*1 (Fig. 2B).

**Fig 3:**
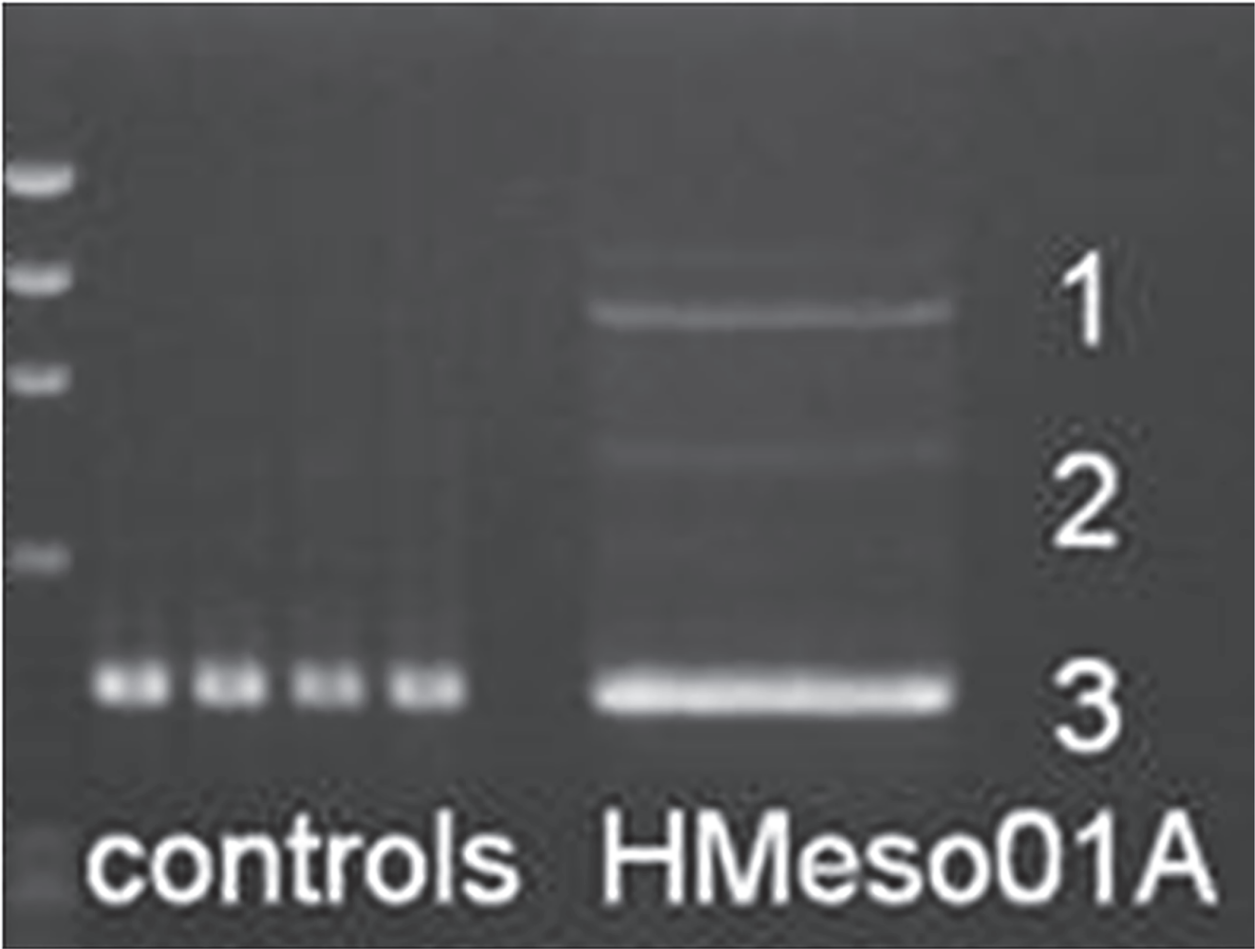
Detection of aberrant splicing products in HMeso01A harboring the *BAP1 c*.*2054 A>T* (p.E685V) mutation. PCR fragments generated from amplification of *BAP1* exons 14–17 on cDNAs from HMeso01A and 4 controls were separated by agarose gel electrophoresis. Marker: DNA marker PhiX 174-HaeIII digest ladder.

To evaluate the effect of the *BAP1 c*.*2054 A>T* (p.E685V) mutation on RNA splicing, we amplified *BAP1* cDNA from four *BAP1* wild type patients and the mutant c.2054 A>T HMeso01A cell line that was derived from a malignant pleural mesothelioma patient. PCR was designed to generate fragments that contained exons 14–17, which are most likely affected by this mutation. Upon separation of the PCR products, HMeso01A cell line displayed aberrant splicing products not observed in the four wild type controls (Fig. 3).

**Fig 4:**
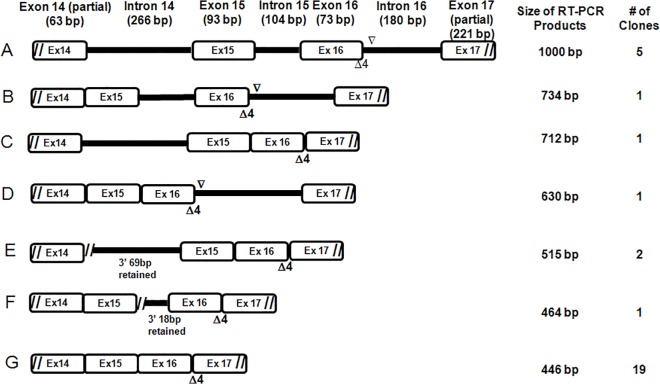
*BAP1 c*.*2054 A>T* (p.E685V) mutant creates a novel 5’ splice site that results in a 4 nucleotide deletion of the 3’ end of exon 16. A: Sequences adjacent to the *BAP1 c*.*2054 A>T* mutation. The splicing sites for the wild type and mutant sequences are indicated. B: Sequence analysis of the major splicing product in HMeso01A indicated deletion of 4 nucleotides at the end of exon 16.

**Fig 5:**
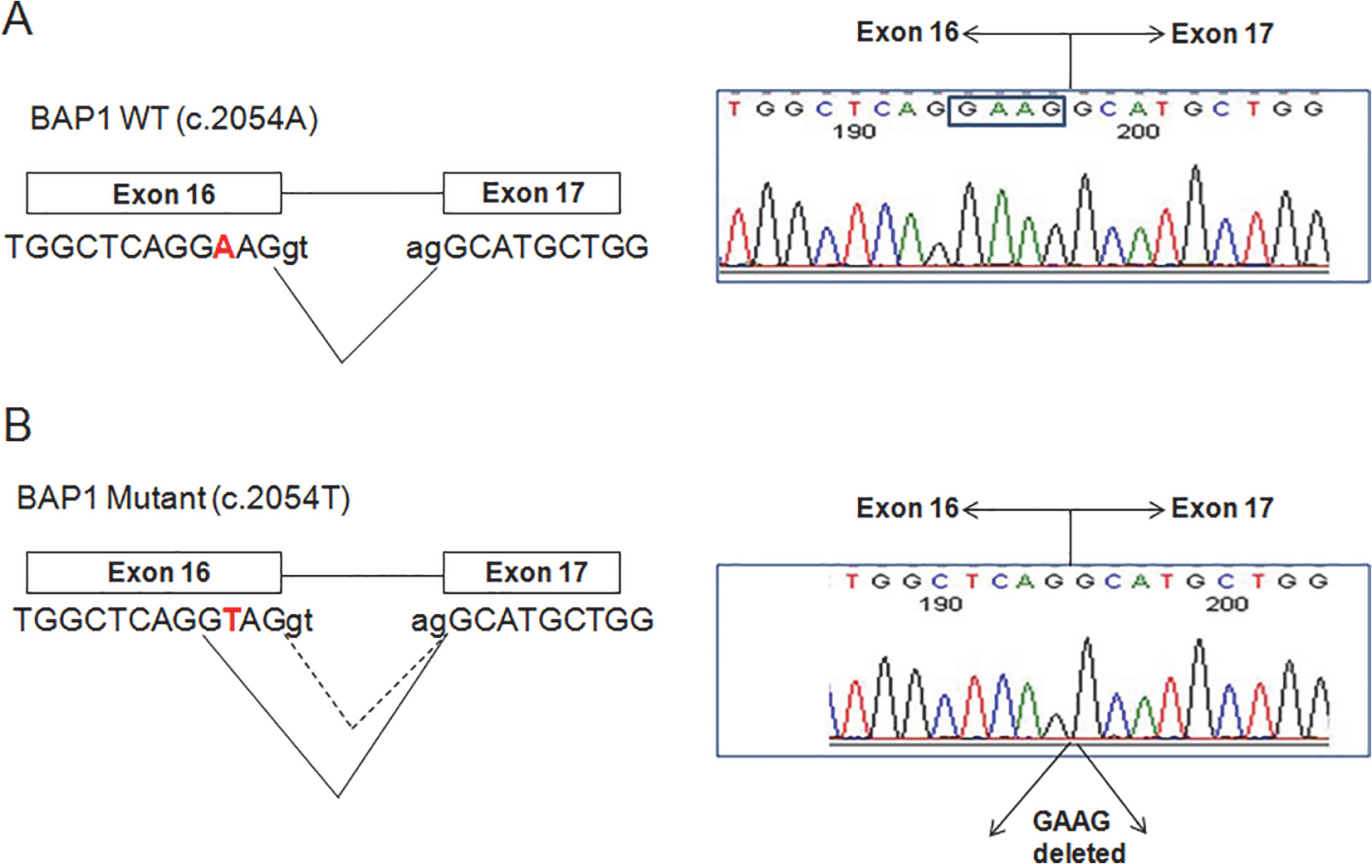
Summary of splicing variants cloned from RT-PCR products of HMeso01A harboring the c.2054 A>T (p.Glu685Val) mutation. A. Retention of introns 14, 15, 16; B. Retention of intron 15, 16; C. Retention of intron 14 and 4 bp deletion (GAAG) at the end of exon 16; D. Retention of intron 16; E. Partial retention of 3’ 69 bp in intron 14 and 4 bp deletion (GAAG) at the end of exon 16; F. Partial retention of 3’ 18 bp in intron 15 and 4 bp deletion (GAAG) at the end of exon 16; G. 4 bp deletion (GAAG) at then end of exon 16 (Δ4). //: partial exon.

We then proceeded to determine whether the *BAP1 c*.*2054 A>T* mutation completely abolishes normal splicing because the bottom band (#3) exhibits similar size as those in the controls. To address this issue, we performed gel extraction of the three major RT-PCR products (1, 2 and 3) from the HMeso01A cell line and cloned them into pCR4 TOPO vectors. PCR amplification and sequencing of *BAP1* exons 14–17 was performed on 30 clones. The cloning results indicate all 19 clones derived from the bottom band in the HMeso01A cell line (Fig. 3) contained a 4 bp deletion compared with the full length transcript. As shown in Fig. 4, the *c*.*2054 A>T* mutation at the 3^rd^ nucleotide from the 3’end of exon 16 introduced a new 5’ splice site (GU), which was recognized by the splicing machinery. The utilization of this newly created splicing site resulted in the deletion of 4 base pairs in the mature cDNA, which presumably leads to a stop codon at amino acid position 690. The mutation presumably produced a truncated BAP1 protein of 689 amino acids instead of 729. In addition, we observed a variety of alternative splicing products that led to retention of different introns (Fig. 5). We observed retention of introns 14, 15 and 16 (the 1000 bp RT-PCR product, note: we can’t completely rule out the possibility that this product might also be generated from traces of genomic DNA contamination in RNA extraction), introns 15 and 16 (the 734 bp product), intron 14 (the 712 bp product) and intron 16 (the 630 bp product). In addition, we also observed partial intronic retentions that generate the 515bp product (with 69 bp of the 3’ end of intron 14 retained) and the 464 bp product (with the 18 bp of 3’end of intron 15 retained). The partial intronic retentions of introns 14 and 15 are caused by activation of alternative 3’ splice acceptor sites (AG) in the introns. Taken together, we were unable to detect any correctly spliced mRNA transcripts in the MPM cell line, HMeso01A, that harbors the missense mutation *BAP1 c*.*2054 A>T* (p.Glu685Val). These results suggest that aberrant splicing caused by this mutation is quite efficient as it completely abolishes normal mRNA splicing in the mutant alleles.

## Discussion

More efficient sequencing technologies are yielding a large number of single nucleotide variations that need to be classified as pathogenic or neutral. Computational methods have been developed to predict the effect of a particular missense mutation, utilizing information on evolutionary conservation and physiochemical properties of amino acid substitutions.[26] However, predictions based solely on statistical differences between wild type and variant amino acid sequences are not yet clinically applicable and the biological effects of missense mutations may remain unclear without mRNA analysis. [14,27]

Bioinformatic analysis predicts that the *BAP1* missense mutation c.2054 A>T (p.Glu685Val) is most likely damaging because it alters a highly conserved amino acid. Interesting, splicing tools predicted it to create a new 5’ splice site. In this study we demonstrate that the c.2054 A>T mutant completely abolishes normal splicing of *BAP1*. It produces multiple splicing variants and, more importantly, creates a new donor site that results in a transcript with a 4 nucleotide deletion in the 3’ end of exon 16. This frameshift mutation presumably results in a premature truncation of the BAP1 protein at amino acid position 690. The mutant allele putatively produces a truncated BAP1 protein of 689 amino acids instead of 729.

Normal mRNA splicing requires accurate recognition of each extremity of each intron by the spliceosome. Introns are identified by the binding of U1 snRNP to the 5’ splice site (5’ss), SF1/BBP to the branch site and the U2AF65/U2AF35 complex to the 3’ splice site (3’ss).

The mammalian U1snRNP complex consists of a 165-nucleotide long molecule (U1 snRNA) and ten different proteins. U1snRNP initiates spliceosome assembly by binding to the 5' ss through base pairing between the single stranded terminal sequence of the U1 RNA molecule and the loosely conserved stretch of nucleotides at the 5' splice site (CAG/GURAGU) which marks the exon-intron boundary. [28] In this 9 nt consensus sequence (which can sometimes be expanded to include 11 base pairs), only the GU dinucleotide is predominantly conserved in the vast majority of the 5'ss that make up the GT-AG introns in mammals. The contribution of other base pairs roughly correlates with their conservation. The consensus -3C forms a C-G base pair with U1, but the conservation of this nucleotide and its contribution to splicing are less important.

[29] Based on a recent collection of 201, 541 human authentic (well-annotated) 5’ss sequences, including 15 nt on each site of the exon/intron junction, the conservation, i.e. the frequency of four nucleotides, at the -3 position is C>A>G>U. [30] As predicted in Fig. 2B, the mutation not only disrupts the consensus 5’ ss motif, but more importantly, creates a new 5’ splice site that matches the CAG/GURAGU which is even stronger than the authentic site. Our splicing analysis demonstrates that this new splice site is indeed utilized by the splicing machinery, which leads to a 4 nucleotide deletion in the 3’ end of exon 16.

Since the splice-site consensus sequences exist and are degenerate, many matches to each consensus are present along pre-mRNAs. Cryptic splice sites, by definition, are sequences that match the consensus motif, but are not detectably used in wild-type pre-mRNA. They are only selected as a result of a mutation elsewhere in the gene, most often at the authentic splice site. [31] We believe the abnormal splicing products detected in this study resulted from the activation and utilization of cryptic splice sites present in the neighboring introns. Since our RT-PCR primers sit in exons 14 and 17, it is reasonable to speculate that there might be other abnormal splicing products that went undetected by this study due to the fact that they lie outside the boundaries of the amplified region.

## Conclusions

In conclusion, this study demonstrates that mutations outside of the highly conserved GU/AG splice sites may disrupt normal splicing instead of creating an amino acid substitution in the mature protein. Therefore, the potential disruption of normal mRNA splicing needs to be considered for exonic substitution variants of unknown significance, both missense and synonymous.
